# Mechanisms and time-resolved dynamics for trihydrogen cation (H_3_^+^) formation from organic molecules in strong laser fields

**DOI:** 10.1038/s41598-017-04666-w

**Published:** 2017-07-05

**Authors:** Nagitha Ekanayake, Muath Nairat, Balram Kaderiya, Peyman Feizollah, Bethany Jochim, Travis Severt, Ben Berry, Kanaka Raju Pandiri, Kevin D. Carnes, Shashank Pathak, Daniel Rolles, Artem Rudenko, Itzik Ben-Itzhak, Christopher A. Mancuso, B. Scott Fales, James E. Jackson, Benjamin G. Levine, Marcos Dantus

**Affiliations:** 1 Department of Chemistry, Michigan State University, East Lansing, Michigan 48824 USA; 2 J. R. Macdonald Laboratory, Department of Physics, Kansas State University, Manhattan, Kansas 66506 USA; 3 Department of Physics and Astronomy, Michigan State University, East Lansing, Michigan 48824 USA

## Abstract

Strong-field laser-matter interactions often lead to exotic chemical reactions. Trihydrogen cation formation from organic molecules is one such case that requires multiple bonds to break and form. We present evidence for the existence of two different reaction pathways for H_3_^+^ formation from organic molecules irradiated by a strong-field laser. Assignment of the two pathways was accomplished through analysis of femtosecond time-resolved strong-field ionization and photoion-photoion coincidence measurements carried out on methanol isotopomers, ethylene glycol, and acetone. *Ab initio* molecular dynamics simulations suggest the formation occurs via two steps: the initial formation of a neutral hydrogen molecule, followed by the abstraction of a proton from the remaining CHOH^2+^ fragment by the roaming H_2_ molecule. This reaction has similarities to the H_2_ + H_2_^+^ mechanism leading to formation of H_3_^+^ in the universe. These exotic chemical reaction mechanisms, involving roaming H_2_ molecules, are found to occur in the ~100 fs timescale. Roaming molecule reactions may help to explain unlikely chemical processes, involving dissociation and formation of multiple chemical bonds, occurring under strong laser fields.

## Introduction

The study of laser-matter interactions using intense laser fields has been an active area of research since the emergence of femtosecond lasers and has led to the discovery of several interesting phenomena^1^. Due to its relative simplicity, the atomic response to strong optical fields is fairly well understood compared to that of molecules. However, some pioneering studies have greatly increased our knowledge of how polyatomic molecules behave in strong laser fields by investigating phenomena such as field induced alignment^2–4^, enhanced ionization^5, 6^, and Coulomb explosion^7, 8^. It is known that strong-field laser-molecular interactions lead to reactions involving multiple bond cleavage and formation processes, and the relative yields of such reactions can be manipulated to some extent by tailored femtosecond pulses^9, 10^.

First discovered by J. J. Thomson in the early 20^th^ century^11^, trihydrogen cations are considered as the simplest and most abundant tri-atomic ions in the universe. Formed via an efficient ion-neutral bi-molecular reaction^12^, H_2_^+^  + H_2_ → H_3_^+^  + H, trihydrogen cations can be commonly found in hydrogen plasmas. Following its spectroscopic identification^13^, H_3_^+^ has been found to be abundant in many atmospheric and interstellar media, such as in the molecular-gas rich Central Molecular Zone (CMZ) and the ionosphere of the three gas giants in our planetary system, where it plays an unparalleled role in interstellar gas-phase chemistry. The formation of H_3_^+^ starting from organic molecules is a unique chemical reaction as it requires cleavage and consecutive formation of three bonds. Such exotic reactions carry a significant importance in photochemistry and molecular physics as they provide an understanding of intramolecular mechanisms at a fundamental level. In small molecules, there has been evidence of hydrogen atom migration on ultrafast time scales as well as the production of trihydrogen cations from a number of organic molecules^14^. Multiple bond cleavage and formation in organic molecules in the process of creating H_3_^+^ have been studied in methanol isotopomers using electron impact^15, 16^, highly-charged ion collisions^17, 18^, and intense laser fields^19–21^. Despite the existence of such studies, which have explored this reaction in detail, uncertainties still exist, particularly regarding the mechanism(s) and timescales for H_3_^+^ formation, which have been reported as ranging from a few picoseconds^19, 20^ up to a few tens of picoseconds^22^.

**Figure 1:**
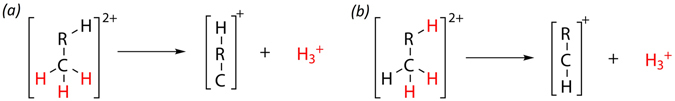
Two formation pathways for H_3_^+^ from a doubly ionized precursor state CH_3_RH^2+^. (**a**) Association of three hydrogen atoms initially attached to the same carbon atom. (**b**) Association of two hydrogen atoms bound to a carbon atom with a hydrogen atom from an adjacent group. The hydrogen atoms that take part in H_3_^+^ formation are indicated in red.

Taking into account the importance of having a definitive understanding of H_3_^+^ formation dynamics in polyatomic molecules under strong-field conditions, we experimentally and theoretically consider H_3_^+^ formation from organic molecules via two distinct pathways following double ionization of the precursor molecule: (i) association of three hydrogen atoms initially bound to the same carbon atom [Fig. 1(a)], and (ii) association of two hydrogen atoms bound to a carbon atom with a hydrogen atom from a neighboring chemical group [Fig. 1(b)]. In each pathway, H_3_^+^ formation occurs via a two-step mechanism in which first the neutral H_2_ moiety is formed from the doubly charged precursor state, i.e. CH_3_OH^2+^, and then abstracts a third proton from the remaining CHOH^2+^ fragment, either from the carbon atom or from the hydroxyl group, forming a triatomic hydrogen cation. Hence, we explore two types of precursor molecules: those containing three hydrogens bound to a single carbon atom (i.e. methyl group), as well as those containing only two hydrogen atoms bound to a carbon atom containing a hydroxyl group.

The first formation pathway has been proposed to follow multiple ionization of methanol isotopomers under strong laser fields^19–21^, and it was shown that H_3_^+^ formation could occur prior to or subsequent to an exchange of the hydrogen atom bound to the hydroxyl group with a methyl hydrogen, and could take up to a few picoseconds^19, 20^. Recent work has addressed the existence of these two mechanisms proposed in refs 19 and 20 by taking advantage of two-color strong-field asymmetry^23, 24^. Hydrogen migration in deuterated methanol (CH_3_OD) under the influence of an asymmetric two-color femtosecond laser field confirmed the existence of the two mechanisms (formation of H_3_^+^, H_2_D^+^), however, both pathways showed the same asymmetry and this led to the interpretation that deuterium migrated to the carbon atom prior to H_2_D^+^ formation^23^. Furthermore, no direct comparison has been made between formation times, nor has the branching ratio between the two pathways been examined under strong-field conditions. The presence of two pathways under electron impact ionization of methanol has been investigated by coincidence momentum imaging and showed that the first formation pathway is four times more favorable than the second^15^. However, due to differences in ionization and fragmentation dynamics under femtosecond ionization compared to those under electron ionization^25^, such conclusions may not be directly applicable to strong-field ionization chemistry.

In the present work, the two formation pathways were studied by dissociative ionization of several organic molecules, including methanol isotopomers (CH_3_OH, CH_3_OD, CD_3_OH, and CD_3_OD), ethylene glycol (C_2_H_6_O_2_), and acetone (C_3_H_6_O) using time-of-flight mass spectrometry and photoion-photoion coincidence (PIPICO) measurements^26–28^. To accurately determine the formation times for each pathway, we conducted pump-probe time-resolved measurements with femtosecond temporal resolution^29^. Please see the Methods: Experimental Setup section for additional details. In conjunction, we have performed *ab initio* molecular dynamics calculations to support our findings. Please refer to the Methods: Computational Details section for information regarding calculations.

## Results and Discussion

**Figure 2:**
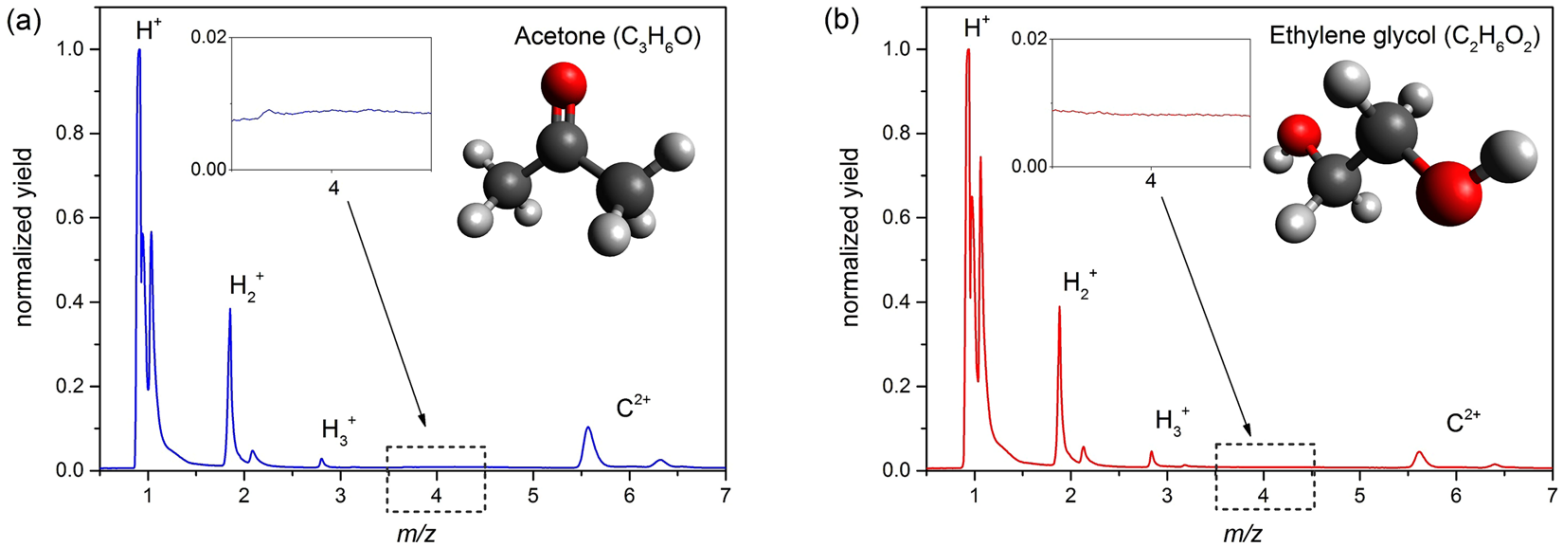
Truncated time-of-flight ion spectrum for (**a**) acetone and (**b**) ethylene glycol in a linearly polarized laser focus of 2.5 × 10^14^ W/cm^2^ under identical experimental conditions. Only ions relevant to this discussion (i.e. with a mass-to-charge ratio *m/z* < 7) are shown. Each yield is normalized with respect to the peak value of the corresponding H^+^ yield. Note that in both spectra, no C^3+^ at *m/z* = 4 was observed (see corresponding inset). The higher yield for forward (early time) ion signals is caused by an extraction slit as explained in the Methods: Experimental Setup section.

In order to investigate the two H_3_^+^ formation pathways, we obtained time-of-flight mass spectra for two distinct organic molecules: one having two methyl groups without a hydroxyl group (acetone) to confirm the first formation pathway and the other with two hydroxyl groups without having a methyl group (ethylene glycol) to confirm the second formation pathway. Figure 2 presents the mass spectra for acetone and ethylene glycol molecules upon irradiation by an intense laser field at 2.5 × 10^14^ W/cm^2^. The peak at *m/z* = 3 is identified as H_3_^+^ yield. However, due to the degeneracy in *m/z* of H_3_^+^ and C^4+^ it is imperative to confirm that there is no contribution from C^4+^ to the ion yield at *m/z* = 3. A close examination of the time-of-flight yield at *m/z* = 4 (see Fig. 2 insets) indicates that there are no events recorded which could be attributed to C^3+^, an expected precursor to the formation of C^4+^. Therefore, it is evident that there is no possibility to produce C^4+^ under these experimental conditions.

The absence of other H_3_^+^ formation pathways from either of the two molecules can be intuited from their molecular structures (shown in Fig. 2). In ethylene glycol, one group of methylene hydrogens is furthest from the other group of methylene hydrogens [diagram in Fig. 2(b), shown in anti-configuration but applicable to other configurations], making the H_3_^+^ formation via association within the two methylene groups unfeasible. In the case of acetone, however, [1,3] hydrogen migration that is followed by an associative H_3_^+^ formation could be considered given the applicability of [1,3] H^+^ migration in acetone enolate^30^. In the current work, however, where intense femtosecond pulses are employed, electrons are initially abstracted within the optical cycles of the electric field, leaving a multiply-charged parent ion^31^. In this condition, the positive charge build up on the oxygen atom draws electronic density from the methyl groups to be localized mainly within the carbonyl group, which leaves the terminal methyl groups at elongated separations through which the [1,3] migration becomes less favorable compared to the associative H_3_^+^ formation from one methyl group. Therefore, observing H_3_^+^ from acetone dissociative ionization confirms the existence of the first formation pathway, association of three hydrogen atoms from the methyl group. Observation of H_3_^+^ from ethylene glycol, confirms the existence of the second formation pathway, association of two methylene hydrogens with the hydrogen atom from a neighboring group.

To further investigate the two pathways for H_3_^+^ formation under identical conditions, an organic molecule capable of reacting via both pathways has to be studied. As methanol (CH_3_OH) contains both a methyl group and a hydroxyl group, CH_3_OH and its isotopomers, CH_3_OD, CD_3_OH, and CD_3_OD, were used in this study to identify the two pathways accurately.

**Figure 3:**
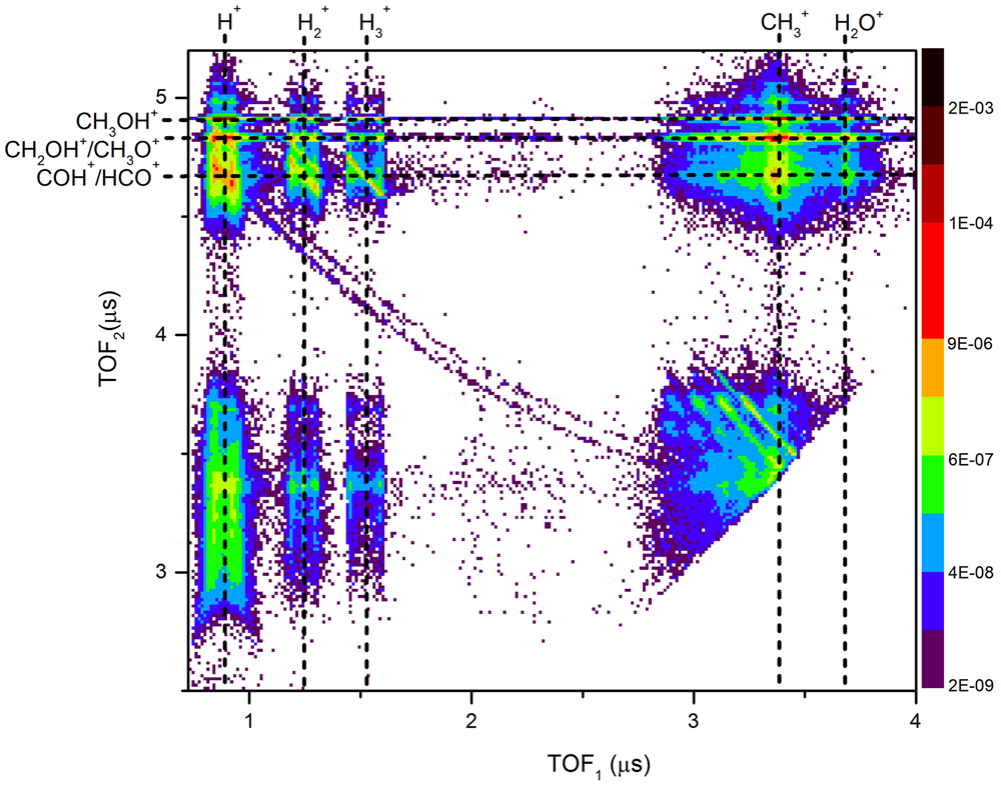
PIPICO map from dissociative ionization of CH_3_OH. On the PIPICO map, the five vertical (dashed) lines represent the approximate center lines of the regions where H^+^, H_2_^+^, H_3_^+^, CH_3_^+^, and H_2_O^+^ ions are recorded. The three horizontal (dashed) lines indicate CH_3_OH^+^, CH_2_OH^+^/CH_3_O^+^, and COH^+^ /HCO^+^ ion regions. The contour region with an approximate slope of −1 at the intersection of the vertical H_3_^+^ line and the horizontal COH^+^/HCO^+^ line represents the coincidence channel of H_3_^+^and COH^+^/HCO^+^. The logarithmic color scale depicts the event rate in units of events/shot.

First we obtained a PIPICO map from dissociative ionization of CH_3_OH in a laser field of 5.0 × 10^14^ W/cm^2^. Figure 3 presents the PIPICO map for photoionization of CH_3_OH (see Supplementary Information Fig. S1 for corresponding time-of-flight mass spectrum). Observing H_3_^+^ in coincidence with COH^+^/HCO^+^ provides evidence for H_3_^+^ formation under strong-field dissociative ionization of CH_3_OH. The integrated yield for the pair coincidence channel of H_3_^+^  + COH^+^/HCO^+^ is 3.3 × 10^−3^ events/shot. However, due to the degeneracy in *m/z* of COH^+^ and HCO^+^, it is not possible to distinguish between the two formation pathways from the CH_3_OH isotopomer.

**Figure 4:**
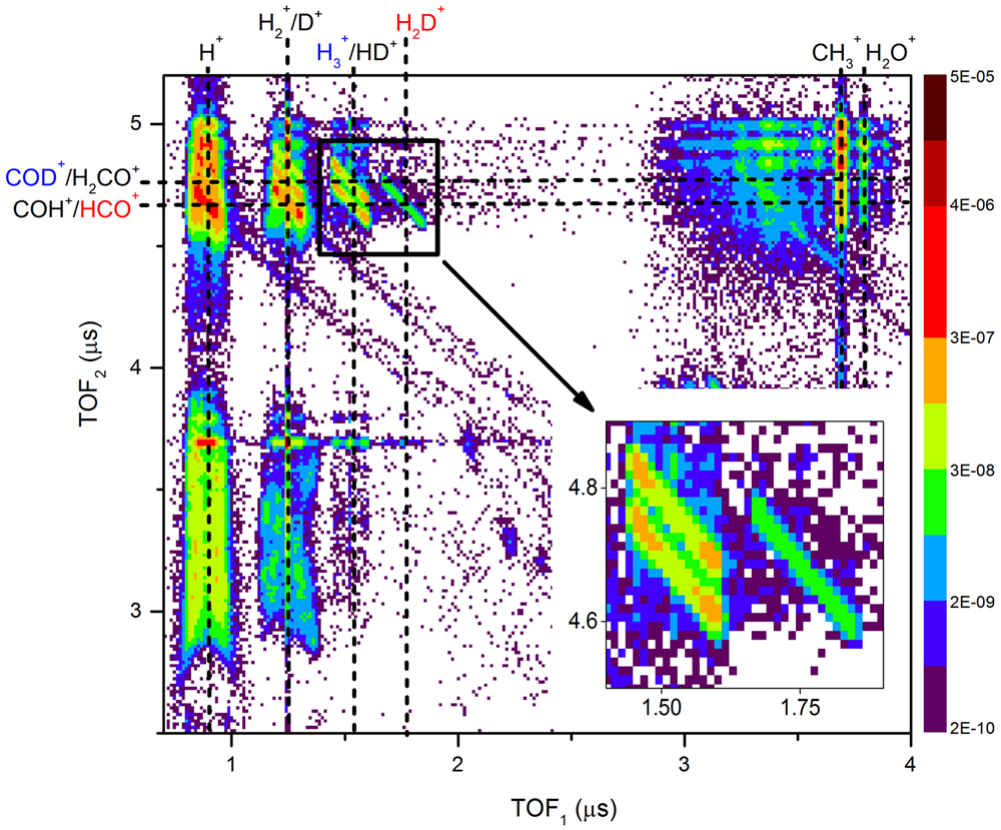
PIPICO map from dissociative ionization of CH_3_OD. On the PIPICO map, the six vertical (dashed) lines represent the approximate center lines of the regions where H^+^, H_2_^+^/D^+^, H_3_^+^/HD^+^, H_2_D^+^, CH_3_^+^, and H_2_O^+^ ions are recorded. The top horizontal (dashed) line indicates the COD^+^/H_2_CO^+^ ion region and the bottom horizontal line indicates the COH^+^/HCO^+^ ion region. The contour region with an approximate slope of −1 at the intersection of the vertical H_2_D^+^ line and the horizontal COH^+^/HCO^+^ line represents the coincidence channel of H_2_D^+^  + HCO^+^ (red colored label) while the contour region at the intersection of the vertical H_3_^+^/HD^+^ line and the horizontal COD^+^/H_2_CO^+^ line represents the coincidence channel of H_3_^+^  + COD^+^ (blue colored label). The coincidence channel of H_3_^+^  + COH^+^/HCO^+^ due to CH_3_OH contamination is visible as the contour region at the intersection of the vertical H_3_^+^/HD^+^ line and the horizontal COH^+^/HCO^+^ line. A magnified view of these three channels is given in the inset. The logarithmic color scale depicts the event rate in units of events/shot.

Having the evidence of H_3_^+^ formation, we continued our investigation using a methanol isotopomer, CH_3_OD, in order to differentiate the pathways. Figure 4 presents the PIPICO map for dissociative ionization of CH_3_OD in a laser field of 6.0 × 10^14^ W/cm^2^ (see Supplementary Information Fig. S2 for corresponding time-of-flight mass spectrum). Here we limit the discussion to three coincidence channels important to our objective (see inset in Fig. 4). Two of them arise from ionization followed by dissociation of the CH_3_OD sample. Specifically, the first two-body breakup channel is H_3_^+^ measured in coincidence with COD^+^, and the second is H_2_D^+^ measured in coincidence with HCO^+^. Due to *m/z* degeneracy, it is possible that the first formation channel contains some coincidence events from the dissociation channel HD^+^  + H_2_CO^+^. The prominent third coincidence channel observed, which is identified as the H_3_^+^  + COH^+^/HCO^+^ channel, is due to a CH_3_OH contamination, as the measurement for CH_3_OD was performed immediately after the CH_3_OH measurements. The integrated yield for the H_3_^+^ channel measured in coincidence with COD^+^ has 2.3 × 10^−4^ events/shot, while the second channel, H_2_D^+^  + HCO^+^, has 4.5 × 10^−5^ events/ shot. An approximate event ratio of 5 to 1 for the two H_3_^+^  + COD^+^ and H_2_D^+^  + HCO^+^ formation channels is evident from these coincident measurements. This indicates that the association pathway of three hydrogen atoms from the methyl group is five times more favorable than H_2_D^+^ formation by association of two hydrogen atoms initially bound to a carbon atom with the deuterium atom from the adjacent hydroxyl group. However, it is worth noting that the branching ratio between the two mechanisms is even higher for the CD_3_OH isotopomer, for which we measured a ratio of about 10 to 1 (see Supplementary Information Figs. S3 and S4), in contrast to previously published results based on electron impact on methanol^15^, which found this ratio to be 4 to 1 for CD_3_OH.

The absence of H_3_^+^ formation pathways starting from a singly-charged parent ion can be intuited from time-of-flight spectra. If H_3_^+^ is formed due to the dissociation of the singly-charged precursor (CH_3_OH^+^), a single peak should be visible at *m/z* = 3, the single peak being characteristic of dissociation into a charged and neutral fragment pair. In contrast, ion-ion repulsion would give rise to a double-peak structure. We only observe such a double-peak structure (see Supplementary Information Fig. S1), where the two peaks are from the forward and backward ejected H_3_^+^ due to the dissociation of the doubly charged precursor (CH_3_OH^2+^). The formation of H_3_^+^ from triply-charged parent cations can be ruled out by closely examining the PIPICO maps for dissociative ionization of CH_3_OH. If H_3_^+^ is formed due to the triply-charged precursor (CH_3_OH^3+^), subsequent to dissociation, a prominent H_3_^+^  + COH^2+^/HCO^2+^ coincidence channel must be visible at the approximate coordinates (1.5 µs, 3.3 µs) on the PIPICO map given in Fig. 3. Furthermore, the absence of the H_2_D^+^  + HCO^2+^ channel at (1.8 µs, 3.3 µs) and the H_3_^+^  + COD^2+^ channel at (1.5 µs, 3.4 µs) in Fig. 4 provides additional evidence that the triply-charged parent cations do not contribute significantly to the formation of H_3_^+^. In addition, we checked for three-body breakup of CH_3_OH, for example, and observed no trace of H^+^  + H_3_^+^  + CO^+^ or H_3_^+^  + C^+^  + OH^+^, therefore supporting the claim that H_3_^+^ is produced following double ionization. However, it is important to note that due to experimental uncertainties, any minute contribution to H_3_^+^ production from singly- or triply-charged precursor states cannot be completely ruled out at our present signal to noise ratio.

Once the two H_3_^+^ formation pathways were identified, we looked at the time-resolved yields for H_3_^+^ formation from CH_3_OH molecules in order to ascertain the formation time.

**Figure 5:**
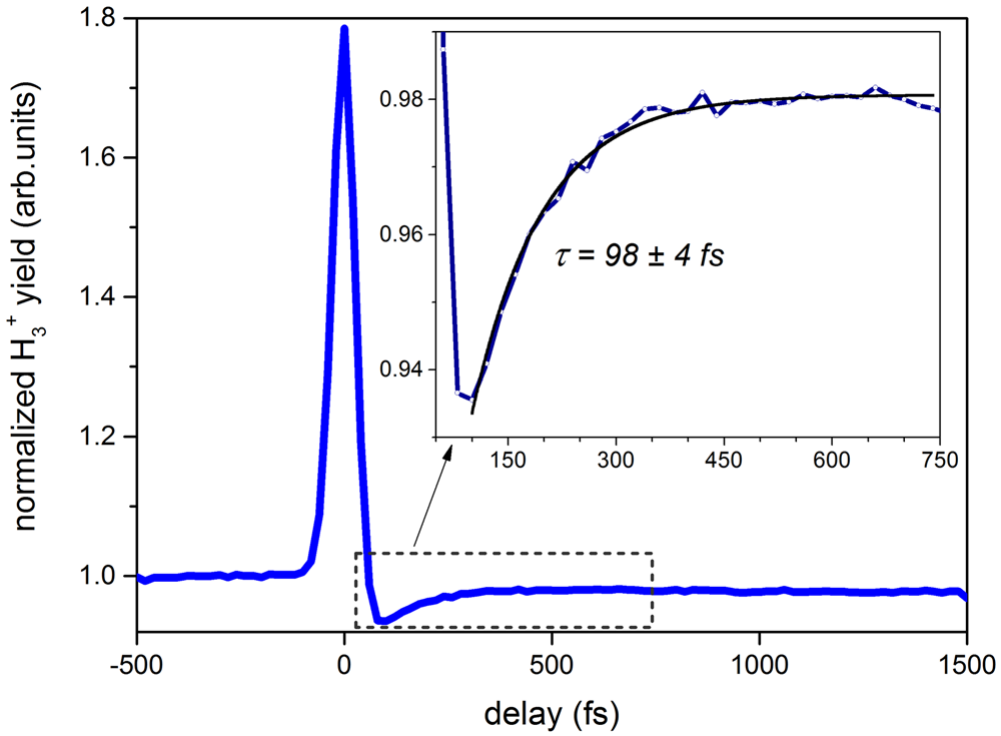
Normalized H_3_^+^ yield (blue solid line) from dissociative ionization of CH_3_OH as a function of applied time delay between the pump and probe pulses. Normalization was performed with respect to the yield at negative time delays. (Inset) Magnified view of the normalized yield in the region of the dashed rectangle is shown together with an exponential fit (black solid line).

Figure 5 presents the time-dependent variation of the H_3_^+^ yield as a function of pump-probe delay over a time period of 1.5 ps. For negative times, the H_3_^+^ yield is independent of time delay. As the pump and probe pulses overlap, the yield reaches a maximum. Once the probe pulse lags behind the pump pulse, the yield goes through a minimum followed by an exponential rise prior to reaching a plateau. This time dependence of the yield can be described as follows. At negative time delays, when the probe pulse arrives earlier than the pump pulse, the formation of the precursor state, i.e. CH_3_OH^2+^, is solely due to the pump pulse. Due to its low intensity, the probe pulse is not capable of forming the precursor by itself, and since it arrives before the pump pulse, it cannot alter the precursor state formed by the pump pulse either. Thus, the H_3_^+^ yield remains constant during negative delays. Once the pulses overlap, at *t* = *0*, the peak intensity of the combined pump and probe pulses increases to a maximum, causing an overall increase in the H_3_^+^ yield. However, when the probe pulse lags behind the pump pulse, the total yield first goes through a minimum before it recovers over a certain time reaching a plateau. The minimum is due to depletion of the precursor (i.e. further ionization and/or fragmentation of the precursor state created by the pump) caused by the probe pulse arriving immediately after the pump. As the arrival time of the probe pulse is further delayed from the pump pulse, the depletion becomes less prominent. The formed H_3_^+^ are minimally disturbed by the probe pulse^32^. Therefore, the time scale for the H_3_^+^ yield recovery is related to the lifetime of the precursor state, thus providing a time scale for the formation of H_3_^+^. The corresponding time scale can be extracted from the pump-probe transient by fitting a suitable exponential curve to the rising edge of the curve. Here we used a fit given by *y* = *y*_*0*_ + *A* exp(*−t/τ*) where *A* is the amplitude, *y*_*0*_ is the offset, and *τ* is the time constant. For the curve given in the inset of Fig. 5, the time constant is *τ* = 98 ± 4 fs, assuming a 95% confidence level for fit parameters. This indicates a fast H_3_^+^ formation time, on the order of 100 femtoseconds, in contrast to previous studies^19, 20^, in which the formation lifetimes of the trihydrogen molecular ions were estimated indirectly through the anisotropy in the measured angular distributions of fragment ions. It is important to keep in mind that the H_3_^+^ yield measured here has contributions from both formation pathways. It is worth noting that an attempt to determine the formation times of the two pathways through a bi-exponential fit was not successful. The most likely reason for this is that the formation times only differ by ~50–100 fs, therefore, the ~40-fs time resolution of the experimental setup leads to a non-converging bi-exponential fit. However, as H_3_^+^ formation via the first channel is more favorable than the second, the time constant given above will more accurately represent the dominant channel.

**Figure 6:**
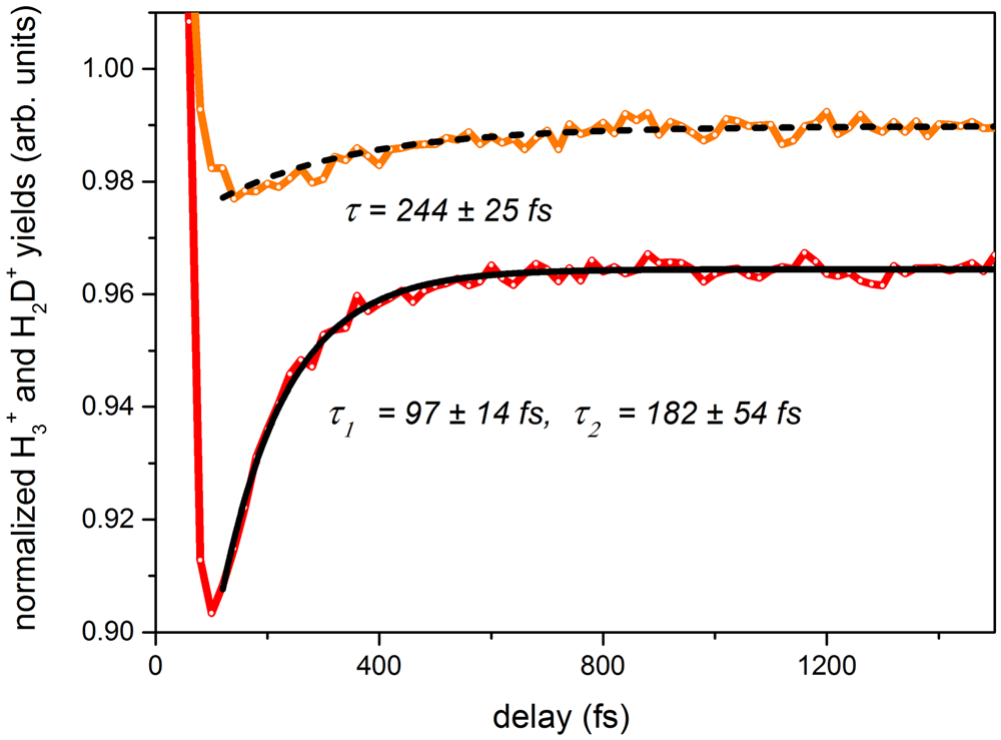
Normalized H_3_^+^ (red solid line) and H_2_D^+^ (orange solid line) yields from dissociative ionization of CH_3_OD as a function of pump-probe delay. Normalization was performed with respect to the yields at negative time delays (see Fig. 5). Exponential fits corresponding to the H_3_^+^ and H_2_D^+^ yields are shown by black solid and dashed lines, respectively.

In order to obtain timing information for both H_3_^+^ formation channels, we studied the dissociative ionization of the methanol isotopomer, CH_3_OD. Figure 6 presents the time-resolved yield for H_3_^+^ from the first formation channel together with H_2_D^+^ from the second channel. Following an exponential fit described previously, the transient for the H_3_^+^ formation channel indicates a time constant of 119 ± 3 fs while the H_2_D^+^ formation channel has a larger time constant of *τ* = 244 ± 25 fs. However, due to *m/z* degeneracy (*m/z* = 3 for H_3_^+^ and HD^+^), the former channel may have an additional contributing channel, i.e. the HD^+^ formation channel. We took this into account by performing a double exponential fit given by *y* = *y*_*0*_ + *A*_*1*_ exp(*−t/τ*_*1*_) + *A*_*2*_ exp(*−t/τ*_*2*_) and found that *τ*_*1*_ = 97 ± 14 fs and *τ*_*2*_ = 182 ± 54 fs with an amplitude ratio of *A*_*1*_ */A*_*2*_ = 4.6. This supports our assumption regarding the existence of an additional (degenerate) channel. By carefully analyzing the ion yields at *m/z* = 1, 2, and 3 for mass spectra from CH_3_OH and CH_3_OD, it was evident that the majority of the yield at *m/z* = 3 for CH_3_OD is due to H_3_^+^ as the contribution from HD^+^ is minor. Therefore, by considering the amplitude difference, *A*_*1*_ > *A*_*2*_, it is evident that the channel indicated by the subscript 1 manifests the formation of H_3_^+^. The time constants for H_3_^+^ formation obtained from CH_3_OH and CH_3_OD are identical within the measurement uncertainties and 2.5 times faster than the time constant for H_2_D^+^ formation, indicating that the association of three hydrogen atoms from the methyl group is a faster process compared to the association of two hydrogen atoms bound to the carbon atom with the deuterium atom from the neighboring hydroxyl group. However, the delay in formation time for the second pathway is partly attributed to orientation and an isotope effect^33^, as further discussed in the subsequent section.

**Figure 7:**
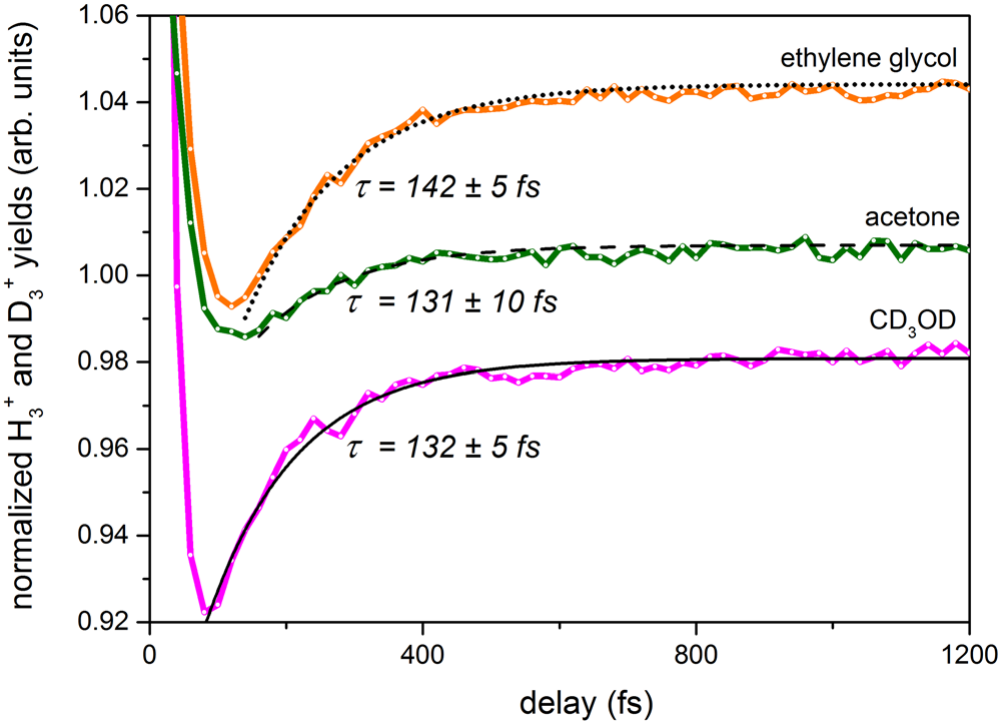
Normalized H_3_^+^ and D_3_^+^ yields from dissociative ionization of different organic molecules as a function of pump-probe delay. Shown in the figure are H_3_^+^ from ethylene glycol (orange solid line), H_3_^+^ from acetone (green solid line), and D_3_^+^ from CD_3_OD (magenta solid line). Normalization was performed with respect to the yields at negative time delays (see Fig. 5). Corresponding exponential fits are shown by black lines.

To further compare the formation times for different H_3_^+^ creation channels and to explore the effect of isotope substitution, we carried out time-resolved measurements for CD_3_OD, acetone, and ethylene glycol (Fig. 7). Following a similar fitting procedure as mentioned before, we obtained time constants for each trihydrogen cation formation channel. For CD_3_OD, the D_3_^+^ formation indicates a time constant of 132 ± 5 fs. For acetone, the time constant for H_3_^+^ formation is 131 ± 10 fs, and for ethylene glycol, it is 142 ± 5 fs. The effect of isotope substitution on formation time is apparent when making a direct comparison between the time constants obtained for CH_3_OH and CD_3_OD, as the time constant for the latter shows a ~34% increase. For acetone, the time constant is larger than that of CH_3_OH, even though the more plausible pathway for H_3_^+^ formation is the association of three hydrogen atoms from the methyl group. This could be attributed to differences in the precursor state. In comparison to H_3_^+^ formation times from CH_3_OH and CH_3_OD, the H_3_^+^ yield from ethylene glycol indicates a longer formation time. This is in agreement with our model, as the sole pathway for H_3_^+^ formation from ethylene glycol entails an association of the hydroxyl hydrogen with two methylene hydrogens.

Trihydrogen cation formation times obtained in this study are summarized in Table 1, arranged in ascending order of formation time. The fast formation times correspond to the first pathway involving the three hydrogen atoms bound to the carbon atom, while the last two entries correspond to the slow channels, which comprise the pathway involving the hydrogen from the hydroxyl group associating with two hydrogens from an adjacent carbon atom. The H_2_D^+^ formation from CH_3_OD was found to be the slowest. This is in part because the HCOD^2+^ ion needs to rotate in order to expose the hydroxyl proton to the roaming H_2_ molecule; some slowing could also be due to the heavier deuteron involved. In comparison to that reaction, H_3_^+^ formation from ethylene glycol via formation pathway 2 occurs faster due to the favorable orientation of the hydroxyl protons, which are pointing at the nascent roaming H_2_ molecule.

**Table 1:** Summary of formation times obtained for trihydrogen cation production from methanol isotopomers, acetone, and ethylene glycol.

Molecule	Trihydrogen cation	Formation time (fs)	Primary formation pathway (1,2)	Isotope effect? (Yes/No)
CH_3_OD	H_3_^+^	97 ± 14	1	No
CH_3_OH	H_3_^+^	98 ± 4	1	No
Acetone	H_3_^+^	131 ± 10	1	No
CD_3_OD	D_3_^+^	132 ± 5	1	Yes
Ethylene glycol	H_3_^+^	142 ± 5	2	No
CH_3_OD	H_2_D^+^	244 ± 25	2	Yes

**Figure 8:**
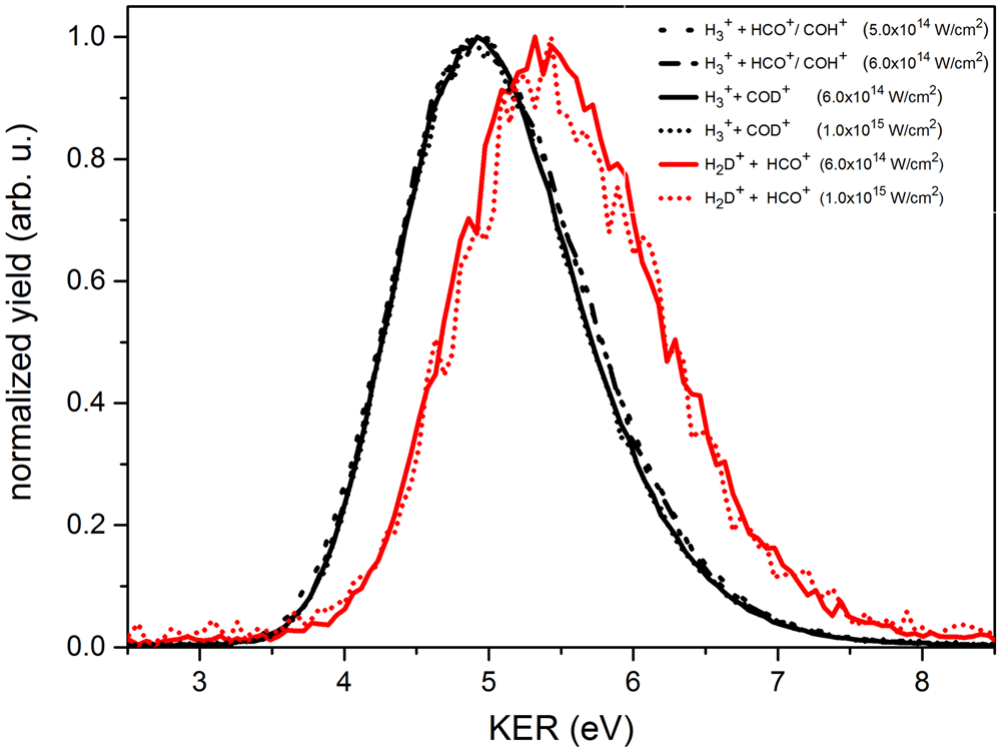
Kinetic energy release (KER) during the formation of trihydrogen cations at different focal intensities for dissociative ionization of CH_3_OH and CH_3_OD. All H_3_^+^ formation channels (black curves) represent a Gaussian distribution with a mean of 5.00 eV and a standard deviation of 0.62 eV, while H_2_D^+^ formation channels (red curves) have a similar distribution with a mean of 5.48 eV and a standard deviation of 0.69 eV.

The kinetic energy release (KER) during a dissociation process provides additional insight into the mechanism^34^. Figure 8 presents the KER distributions calculated from the position and time information of the two ions measured in coincidence following a standard COLTRIMS approach^35, 36^.

It is evident from these KER distributions that regardless of the originating molecule or the laser intensity, similar fragments exhibit almost identical KER. However, a comparison between the H_3_^+^  + COD^+^ and H_2_D^+^  + HCO^+^ dissociation channels from the same precursor molecule, CH_3_OD^2+^, indicates an increase in the KER with the latter pathway (H_2_D^+^ formation). This increase in KER for the H_2_D^+^ formation channel can be attributed, based on conservation of energy and momentum arguments, to the higher thermodynamic stability of the HCO^+^ fragment compared to the COD^+^ fragment. This difference in stability has been predicted to be 1.63 eV at the CCSD(T)/CBS(V + C) + ZPE level of theory for the non-deuterated molecules^37^. Thus, a fraction of this excess energy appears to be converted to additional kinetic energy in the recoiling fragments of the H_2_D^+^  + HCO^+^ dissociation channel. We anticipate a fraction of the remaining energy ends up as internal rotational and vibrational energy of the products. KER analysis confirms that the H_2_D^+^ formation occurs via abstraction of the deuterium atom directly from oxygen, and not through H-migration.

### Theoretical Results

First principles molecular dynamics simulations for the formation of H_3_^+^ from a doubly charged methanol molecule based on the single reference configuration interaction singles and doubles (CISD) method have previously been reported^38^. The CISD trajectories revealed two distinct H_3_^+^ formation pathways. In these pathways, a neutral H_2_ molecule is initially ejected and then later reacts with the CHOH^2+^ to form H_3_^+^ either along with the formyl cation (CHO^+^) or the isoformyl cation (COH^+^). The isoformyl cation formation mechanism, which does not involve the hydroxyl hydrogen in the H_3_^+^ formation, was found to be about an order of magnitude more probable. In our experiments, CHOH^2+^ and CHOD^2+^ were detected in the respective mass spectra (see Supplementary Information Figs. S1 and S2). This observation confirms the ejection of neutral H_2_ from CH_3_OH^2+^. Here we assess the validity of involvement of the neutral H_2_ molecule in the production of H_3_^+^ from the doubly charged methanol.

For our simulations we used the multireference complete active space self-consistent field method with 12 active electrons in 12 active orbitals (CASSCF(12/12)) to investigate the H_3_^+^ formation mechanism. This method is a more flexible treatment of the electronic structure than the single reference CISD method, giving a balanced treatment of regions of the potential energy surface corresponding to closed shell and radical electronic configurations. A summary of the final outcome of the trajectories is provided in Table 2.

**Table 2:** Summary for the final products of the CASSCF trajectories after 150 fs.

Final Products	% Yield
H^+^ + CH_2_OH^+^	48.3
H^+^ + CH_2_OH^+^ (hydroxyl proton ejection)	0.2
H_2_ + CHOH^2+^	18.8
H_2_^+^ + CHOH^+^	23.4
H_2_ + H^+^ + COH^+^	0.1
H_2_ + H^+^ + CHO^+^	5.2
H_3_^+^ + COH^+^	3.9
H_3_^+^ + CHO^+^	0.1

We observe that the ejection of one proton from the methyl side accounts for about half of the trajectories. Moreover, the production of diatomic hydrogen was observed in a large percentage either as neutral H_2_ or cationic H_2_^+^. Interestingly, H_2_^+^ was not predicted in the earlier CISD work^38^, but is observed experimentally in high yield and in coincidence with the formation of CHOH^+^ (Fig. 3). The preferential formation of H_2_^+^  + CHOH^+^ compared to the H_2_  + CHOH^2+^ pathway in the current trajectories is consistent with the fact that the biradical system is more thermodynamically stable by 1.86 eV, as calculated at the complete basis set-atomic pair natural orbital (CBS-APNO) level of theory^37^. In most of the trajectories that form dihydrogen, either neutral or charged, the dihydrogen molecule moves far away from the other fragment, making H_3_^+^ formation impossible. However, when H_3_^+^ formation is observed, it occurs only after the formation of neutral H_2_ in two distinct mechanisms that resemble what was previously found in the early CISD work^38^. In the predominant mechanism, H_2_ roams near CHOH^2+^ and then abstracts a methyl proton to form H_3_^+^ and COH^+^. The 50–130 fs timescale of this process observed in our simulations matches our experimental observations. The second mechanism involves a roaming H_2_ molecule which abstracts a hydroxyl proton to form H_3_^+^ and CHO^+^. This trajectory was observed only once out of 1000 trajectories, and occurred at about 130 fs. The relatively low occurrence of H_3_^+^  + CHO^+^ in our simulations can be explained in part by the fact that the time scale associated with this mechanism is comparable to our total 150 fs simulation window (see Fig. 6). Moreover, a review of the trajectory indicates this pathway depends on the relative distance/orientation of the hydroxyl proton. Our relatively short simulations may not be a good measure of the relative occurrence of this slower mechanism. It is worth noting that the predicted dominance of the formation of H_3_^+^ along with COH^+^ is thermodynamically unfavorable compared to the second pathway (H_3_^+^  + CHO^+^) by 1.63 eV as reported at the CCSD(T)/CBS(V + C) + ZPE level of theory^39^. Yet, the thermodynamic stability of CHO^+^ compared to COH^+^ is clearly reflected upon the ejection of separate H^+^ and H_2_ in our trajectories. The simulations predict a ratio of H_3_^+^ to H^+^ of 7%, which is in good agreement with the experimental mass spectra of methanol.

**Figure 9:**
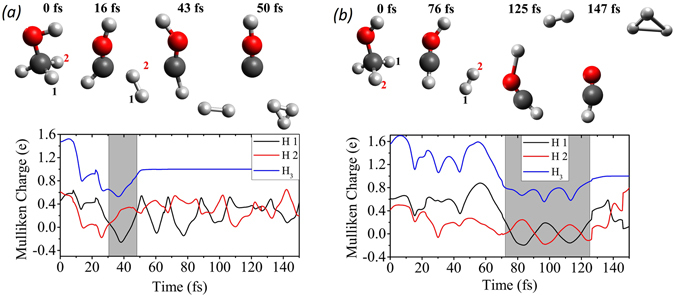
Snapshots from the two trajectories which result in the formation of H_3_^+^ through two different pathways, (**a**) association of three hydrogen atoms initially attached to the same carbon atom and (**b**) association of two hydrogen atoms bound to a carbon atom with a hydrogen atom from a neighboring group. The associated Mulliken charges with each of the H_2_ hydrogen atoms (numbered in the CH_3_OH structure as 1 & 2 and as H 1 & H 2 in the figure legends) as well as the total charge on the nascent trihydrogen cation are shown as a function of time. The grey area highlights the survival time of H_2_ before the formation of H_3_^+^.

Several snapshots from two of the H_3_^+^ formation trajectories are shown in Fig. 9. These two examples represent the two different H_3_^+^ formation pathways. Videos of these two trajectories are provided online as Supplementary Video S1 and Supplementary Video S2. The Mulliken charges for each of the two hydrogen atoms that are forming H_2_ as well as the three hydrogen atoms forming H_3_^+^ are also shown in Fig. 9, to demonstrate that neutral H_2_ roaming is essential for the formation of H_3_^+^. Note that for the second pathway shown in Fig. 9(b), a longer roaming time is observed when the more distant hydroxyl proton is involved.

## Conclusions

The existence, mechanistic details, and timescales of two pathways for trihydrogen cation formation from organic molecules in a strong laser field have been experimentally determined by femtosecond time-resolved time-of-flight mass spectroscopy and photoion-photoion coincidence momentum imaging techniques. Evaluation of coincidence/non-coincidence data together with measured kinetic energy release distributions of the formed trihydrogen cations clearly reveals the existence of two distinct pathways from a doubly charged precursor. The experimental findings indicate that the first pathway, which consists of association of three hydrogen atoms from a methyl group to form the H_3_^+^ cation, is more favorable with a branching ratio up to 10:1 compared to the second channel, where trihydrogen cations are formed via association of two hydrogen atoms bound to a carbon atom with a hydrogen atom from a neighboring chemical group. Furthermore, our femtosecond time-resolved measurements indicate a faster formation time for the first pathway, on the order of 100 fs, compared to the second pathway, which exhibits a formation time twice as long. *Ab initio* molecular dynamics simulations suggest that H_3_^+^ formation occurs via a two-step mechanism in which a neutral H_2_ molecule is formed, and then roams about the doubly charged intermediate until it abstracts a proton from the remaining CHOH^2+^ fragment. Proton abstraction by the roaming H_2_ molecule is reminiscent of the Hogness and Lunn reaction for H_3_^+^ formation in the universe. This mechanism has been shown to play a role in some condensed phase chemical reactions involving superacids^40^, and here we confirm that H_2_ molecule roaming plays an important role in chemical reactions under intense laser fields. Under strong fields, molecules are likely to doubly ionize within a few femtoseconds, these multiply ionized species acting as superacids. The formation of neutral H_2_ molecules is favored by a transition state that places two hydrogen atoms in close proximity. Once formed, neutral H_2_ molecules may roam the ionized intermediate until they extract the nearest proton, the reaction product determined kinetically and not thermodynamically.

## Methods

### Experimental Setup

Two different experimental setups were used to acquire the data for this study. The setup used to acquire the time-of-flight mass spectra consists of a Ti:Sapphire chirped pulse amplification laser system (800 nm, 25 nm FWHM, 1 mJ/pulse, 1 kHz) and a Wiley-McLaren time‐of‐flight mass spectrometer. Note that in our mass spectrometer, the presence of a slit to reduce volume effects causes the yield of fragment ions travelling towards the detector to be higher than those traveling away. No effort was made to calibrate this anisotropic detection because it is not relevant in this study. To obtain time-resolved information, the laser pulse was split into pump and probe pulses in a Mach-Zehnder geometry. More information regarding the setup and data acquisition can be found elsewhere^29, 41^. Residual high-order dispersions in the amplified laser pulses were removed using the multiphoton intrapulse interference phase scan (MIIPS) technique^42, 43^. The transform limited pulse duration was measured to be 38 ± 2 fs. The laser intensity was calibrated by measuring Ar^2+^/Ar^+^ and N_2_^2+^/ N_2_^+^ ion yields^44, 45^ and found to be in agreement with the calculated intensity based on optical measurements within a factor of 2. The base pressure inside the mass spectrometer was kept below 2 × 10^-9^ Torr. Liquid samples were first outgassed using several iterations of freeze-pump-thaw cycles prior to their introduction into the interaction region through an inlet valve creating an effusive beam of gaseous sample. During all measurements, the pressure inside the mass spectrometer while the sample was flowing was kept at or below 2 × 10^−5^ Torr.

A typical time-resolved scan was performed over the range from −500 fs to 1500 fs with a step size of 20 fs. At each time delay, a time-of-flight spectrum was obtained by integrating over 128 laser shots. Each time-resolved plot is an average of several hundred iterations of the applied time delay, thus making each data point presented in this work an average of more than 50,000 laser shots. The polarization of the pump beam was kept parallel to the time-of-flight axis and the intensity was set to 2.5 × 10^14^ W/cm^2^, which provided the best signal-to-noise ratio for the H_3_^+^ yield. The probe intensity, 1.0 × 10^14^ W/cm^2^, was kept well below the threshold to form the precursor state needed for H_3_^+^ formation. Additionally, the polarization of the probe was set to be perpendicular to that of the pump beam. The uncertainty of the measured signal is less than 5%.

The second experimental setup was used to study the reaction products in momentum space and to identify the dissociation channels. This setup consists of a high repetition rate, Ti:Sapphire chirped pulse amplification laser system (PULSAR @ JRML: 10 kHz, 785 nm, 25 fs FWHM, up to 2 mJ/pulse) and a Cold Target Recoil Ion Momentum Spectroscopy (COLTRIMS) setup. Detailed information regarding the COLTRIMS setup, detection, and data acquisition can be found elsewhere^46^. The polarization of the laser beam was set parallel to the time-of-flight axis. Liquid samples were outgassed using the same technique mentioned before and introduced into the chamber through a 40-µm aperture to form a supersonic gas jet. The laser beam was focused onto the skimmed molecular beam by a spherical mirror (*f* = 75 mm) placed inside the UHV chamber, resulting in intensities up to 1.0 × 10^15^ W/cm^2^. The intensity was calibrated by measuring Ne^+^ ion momenta along the laser polarization direction^47, 48^. The pressure in the interaction region when the sample was present was kept below 5 × 10^−10^ Torr to achieve an event rate of less than 1 count/pulse. More than 100 million laser shots were acquired to obtain statistically significant results. It is important to note that the COLTRIMS setup does not utilize a slit to reduce the focal volume effect, and thus produces symmetric backward and forward travelling ion yields in the time-of-flight spectra (Supplementary Figs. S1, S2 and S3) compared to those obtained from the Wiley-McLaren time‐of‐flight mass spectrometer (Fig. 2).

Our first effort was to explore the existence of two different H_3_^+^ formation pathways under strong-field dissociative ionization of organic molecules. Upon identification of such pathways, we performed more in-depth analysis to characterize the identified pathways using coincidence imaging and time-resolved studies. It needs to be emphasized here that due to degeneracy in *m/z* for H_3_^+^ and C^4+^, particularly at high laser peak intensities, proper identification of the H_3_^+^ yield is critical. In all results presented in this work, the H_3_^+^ yield was only considered after careful analysis of adjacent peaks, making sure no C^3+^ was observed and the background was properly subtracted.

### Computational Details

*Ab initio* molecular dynamics simulations of the dissociation of singlet CH_3_OH^2+^ were carried out using the complete active space self-consistent field (CASSCF) method, employing a full valence active space composed of 12 electrons in 12 orbitals. The singlet spin state was chosen both because it is the lowest energy state of methanol dication and because the lowest energy states of the resulting fragments are known to be closed shell singlets. The 6–31G** basis set was used. The initial nuclear positions and momenta were sampled from the vibrational Wigner distribution of the neutral ground state computed in the harmonic approximation at the full valence CASSCF (CASSCF(14/12)/6–31G**) level of theory. This sampling approach approximates the double ionization of neutral methanol as instantaneous. The dynamics were integrated to 150 fs using the velocity Verlet integrator and a time step of 0.5 fs. A total of 1000 trajectories were calculated. Such a large number of trajectories at this level of theory has been achieved with the aid of graphical processing units (GPUs) using a development version of TeraChem^49–52^.

## Electronic supplementary material

Supplementary Video S1

Supplementary Video S2

Supplementary Information
