# Supplementary material for: Mechanisms and time-resolved dynamics for trihydrogen cation (H3+) formation from organic molecules in strong laser fields
Source: Sci Rep. 2017 Jul 5;7:4703. doi: 10.1038/s41598-017-04666-w (PMC5498647; doi:10.1038/s41598-017-04666-w)
Supplement: Supplementary file 3 — Supplementary Information [file 41598_2017_4666_MOESM3_ESM.pdf]

Supplementary Information

Mechanisms and time-resolved dynamics for trihydrogen cation (H_3^+) formation from organic molecules in strong laser fields

Nagitha Ekanayake[1], Muath Nairat[1], Balram Kaderiya[2], Peyman Feizollah[2], Bethany Jochim[2], Travis Severt[2], Ben Berry[2], Kanaka Raju P.[2], Kevin D. Carnes[2], Shashank Pathak[2], Daniel Rolles[2], Artem Rudenko[2], Itzik Ben-Itzhak[2], Christopher A. Mancuso[1], B. Scott Fales[1], James E. Jackson[1], Benjamin G. Levine[1], and Marcos Dantus[1,3 *]

[1] Department of Chemistry, Michigan State University, East Lansing, Michigan 48824, USA.
[2] J. R. Macdonald Laboratory, Department of Physics, Kansas State University, Manhattan, Kansas 66506, USA.
[3] Department of Physics and Astronomy, Michigan State University, East Lansing, Michigan 48824, USA.

** Corresponding author (dantus@chemistry.msu.edu)*

Time-of-Flight Mass Spectra for CH_3OH, CH_3OD, and CD_3OH

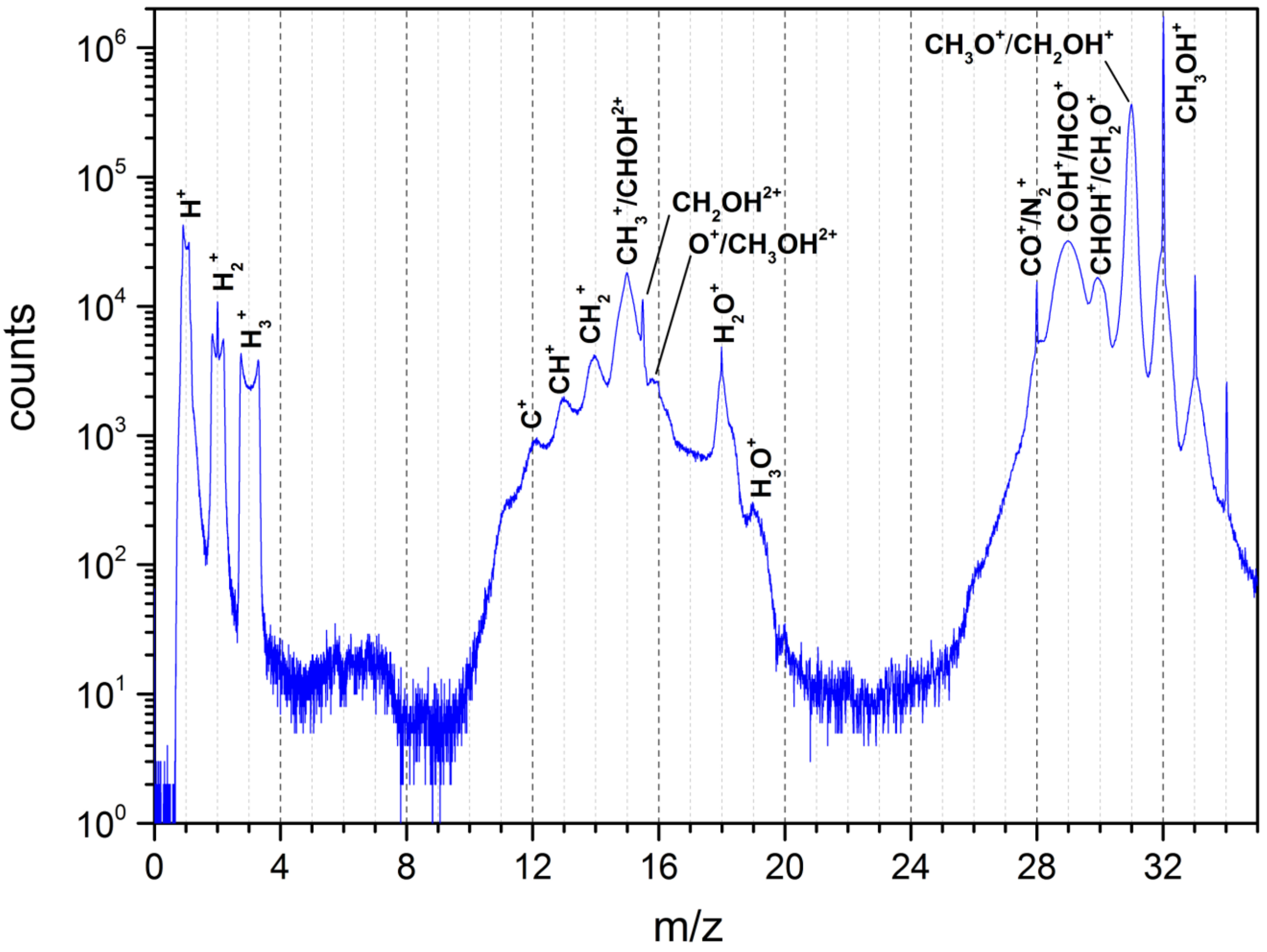

Figure S1. The time-of-flight mass spectrum for dissociative ionization of CH_3OH in a linearly polarized laser focus of 5×10^{14} W/cm^2.

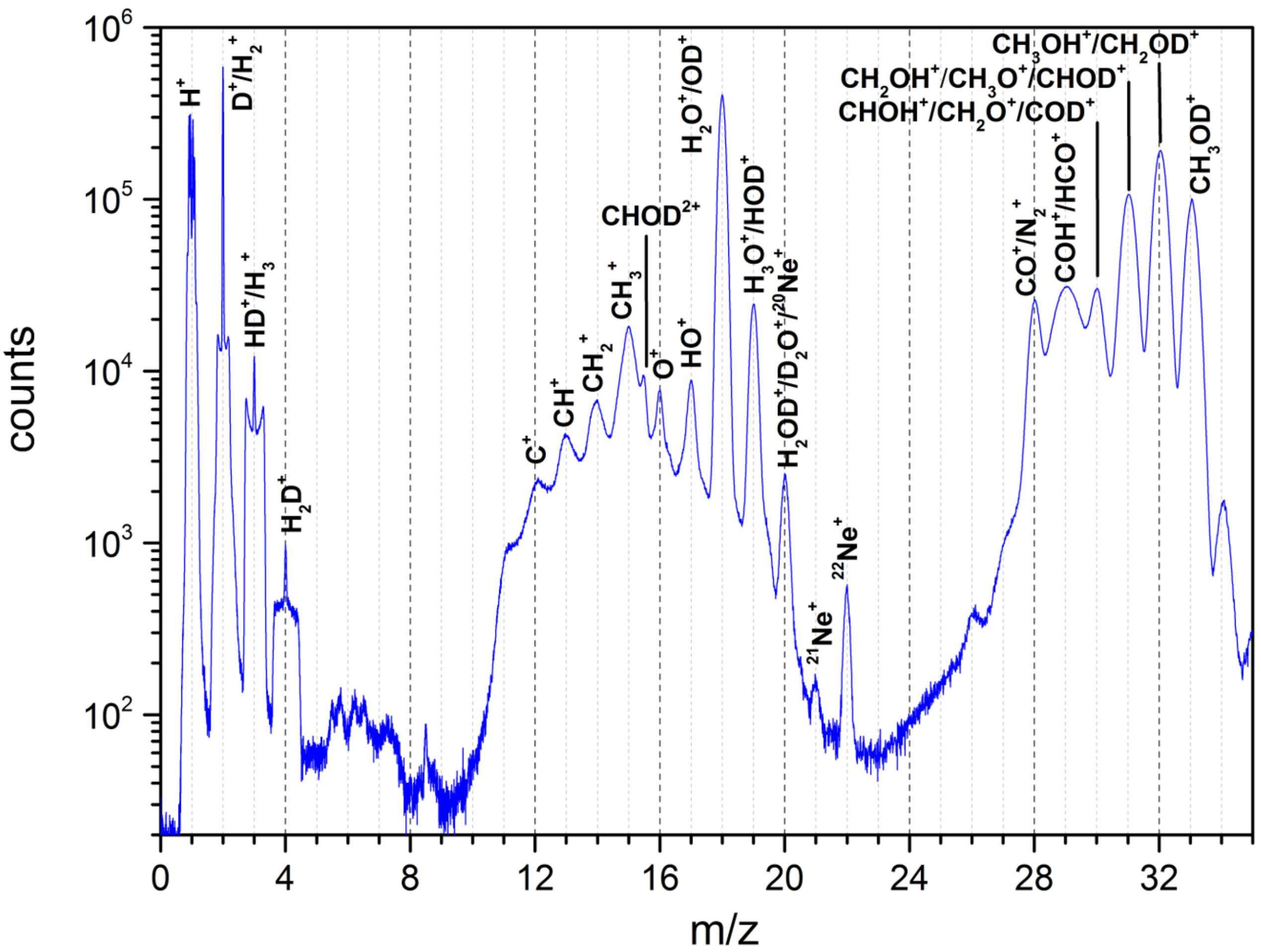

Figure S2. The time-of-flight mass spectrum for dissociative ionization of CH_3OD in a linearly polarized laser focus of 6×10^{14} W/cm^2. As the data acquisition for CH_3OD was performed immediately after the CH_3OH measurements, a CH_3OH contamination is observed in the mass spectrum. The $^nNe^+$ (n =20–22) peak structure arises from the residual neon sample used for intensity calibration.

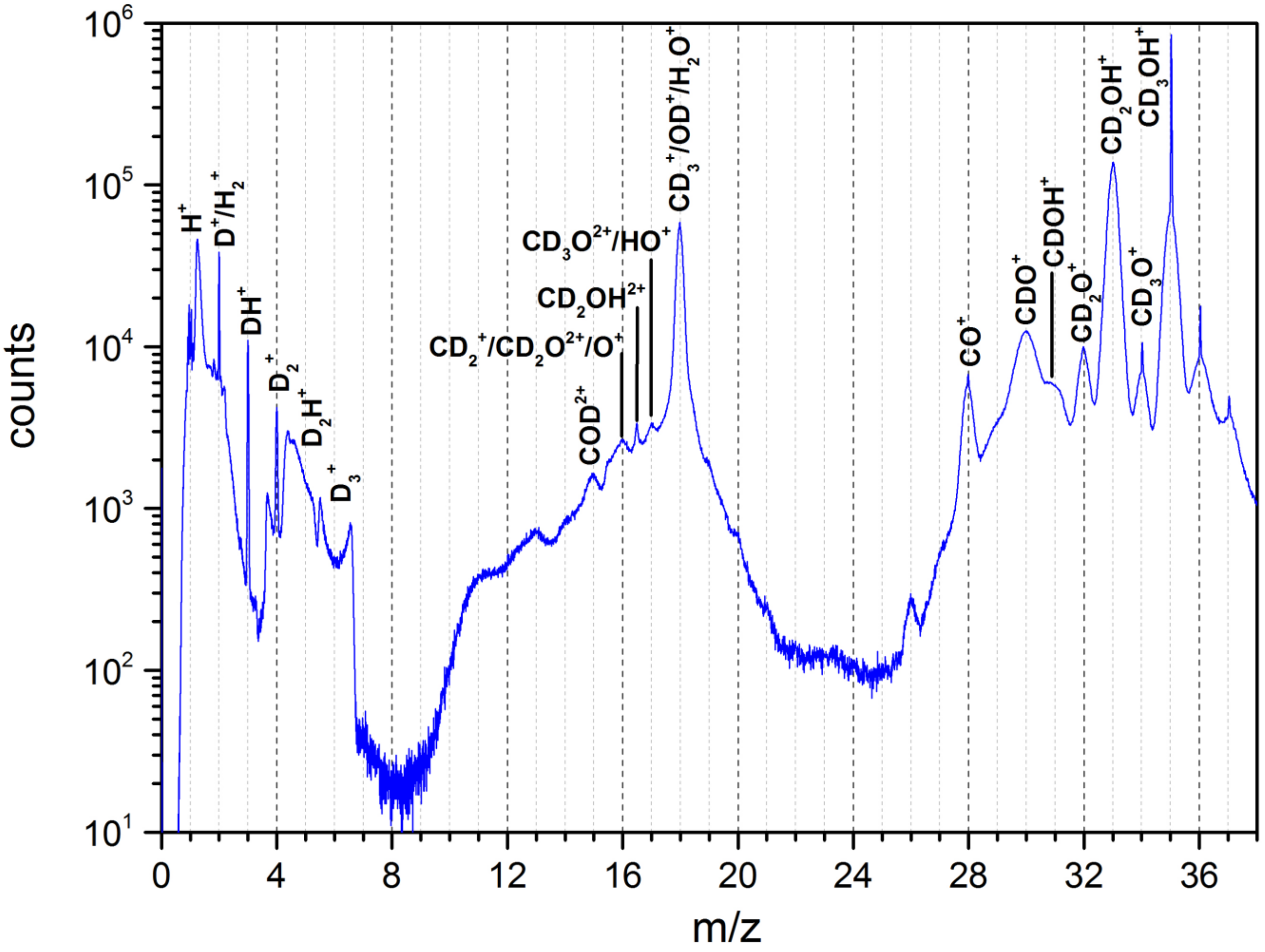

Figure S3. The time-of-flight mass spectrum for dissociative ionization of CD_3OH in a linearly polarized laser focus of 6×10^{14} W/cm^2.

PIPICO Plot for CD_3OH

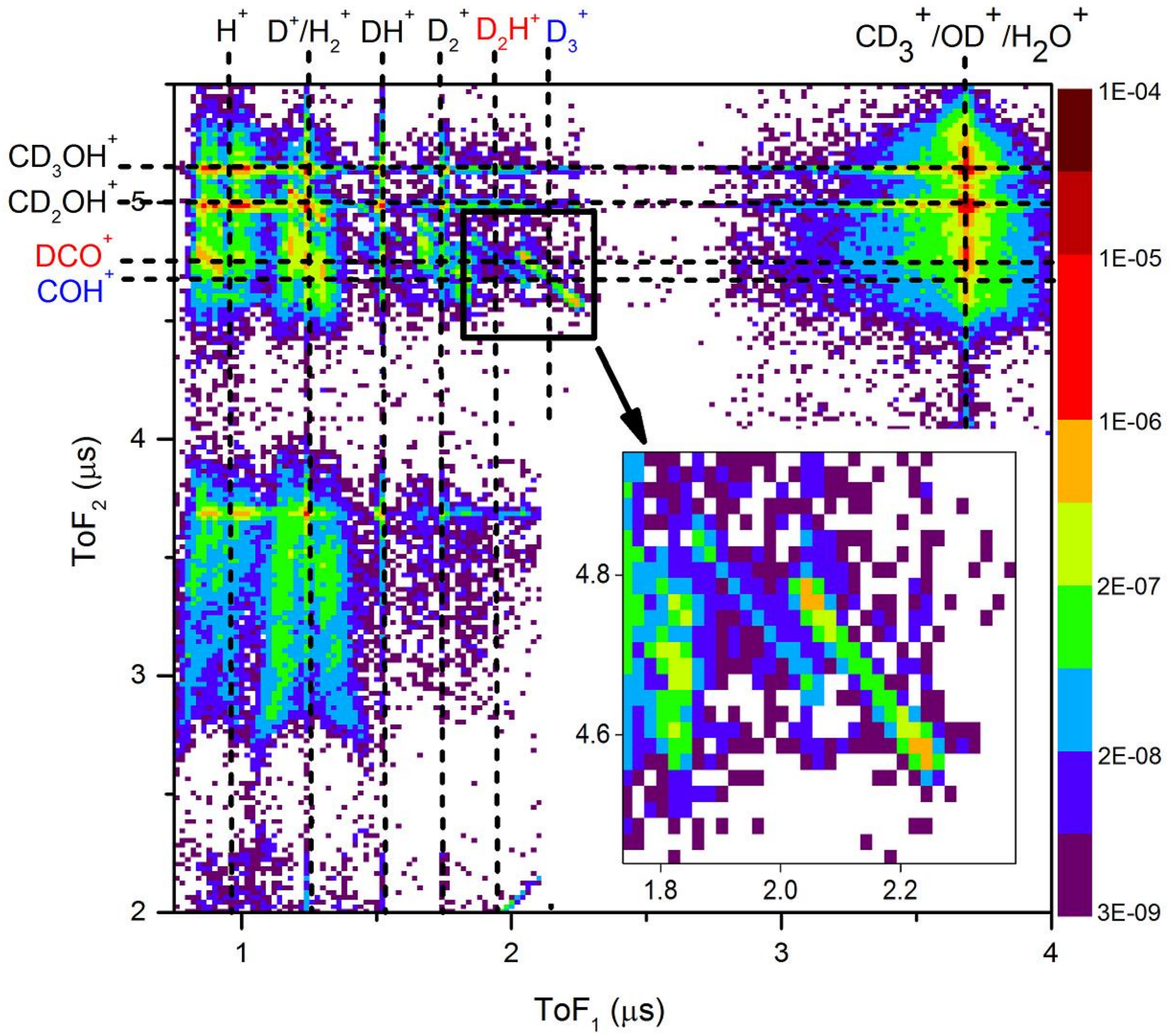

Figure S4. PIPICO map from dissociative ionization of CD_3OH in a linearly polarized laser focus of 6×10^{14} W/cm^2. On the PIPICO map, the seven vertical (dashed) lines represent the approximate center lines of the regions where H^+, D^+/ H_2^+, DH^+, D_2^+, D_2H^+, D_3^+, and $CD_3^+/OD^+/H_2O^+$ ions are recorded. The four horizontal (dashed) lines indicate the approximate center lines of the regions where CD_3OH^+, CD_2OH^+, DCO^+, and COH^+ ions are detected. The contour region with an approximate slope of -1 at the intersection of the vertical D_3^+ line and the horizontal COH^+ line represents the coincidence channel of $D_3^+ + COH^+$ (blue colored label) while the contour region at the intersection of the vertical D_2H^+ line and the horizontal DCO^+ line represents the coincidence channel of $D_2H^+ + DCO^+$ (red colored label). A magnified view of these two channels is given in the inset. The logarithmic color scale depicts the event rate in units of events/shot.The integrated yield for the D_3^+ channel measured in coincidence with COH^+ has 4.3×10^{-6} events/shot, while the second channel, $D_2H^+ + DCO^+$, has 4.4×10^{-7} events/ shot. Thus, an approximate event ratio of 10 to 1 for the two $D_3^+ + COH^+$ and $D_2H^+ + DCO^+$ formation channels is evident.
